# MULTIPLE CUTANEOUS LEIOMYOMAS: PAIN RELIEF WITH PULSED HYSOCINE BUTYL BROMIDE

**DOI:** 10.4103/0019-5154.48994

**Published:** 2009

**Authors:** Feroze Kaliyadan, Jayasree Manoj, A D Dharmaratnam

**Affiliations:** *From Department of Dermatology, Amrita Institute of Medical Sciences and Research Centre, Kochi, Kerala, India*

**Keywords:** *Hyoscine butyl-bromide*, *multiple cutaneous leiomyomas*, *type 2 segmental*

## Abstract

A 35-year-old male patient presented to our outpatient department, complaining of multiple, raised skin lesions on the forehead and back, associated with intermittent pain, especially on exposure to cold. A diagnosis of cutaneous leiomyoma (type 2 segmental) was made, which was confirmed by skin biopsy. The patient was started on a trial of pulsed Hyoscine Butyl bromide tablets, following which the patient had significant relief from pain associated with the lesions.

## Introduction

Leiomyomas are rare benign tumours of skin presenting as solitary or multiple papules and/or nodules.[1,2]

Segmental lesions affect a particular dermatome.[3,4] Cutaneous lesions are usually painful. Varous modalities have been used to relieve pain in such lesions.

## Case Report

A 35-year-old male patient presented to our outpatient department, complaining of multiple raised skin lesions on the forehead and back. The lesions had been gradually increasing in number over the last 10 years. Over the last two years, the patient had started noticing intermittent pain over the lesions, especially on exposure to cold. The patient's father had a history of similar lesions, with pain, restricted to his back. The patient had no other significant co-morbidity.

**Figure 1:**
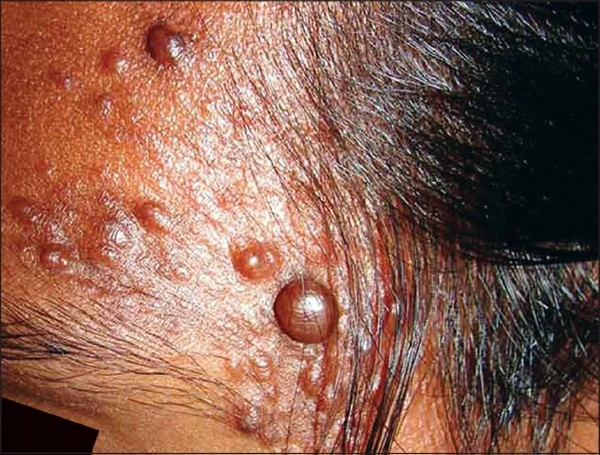
Lesions over the forehead

**Figure 2:**
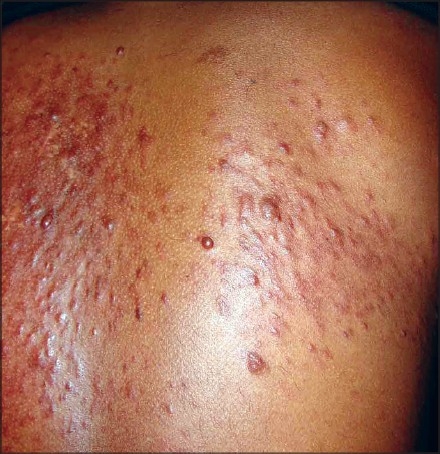
Extensive lesions over the back

**Figure 3:**
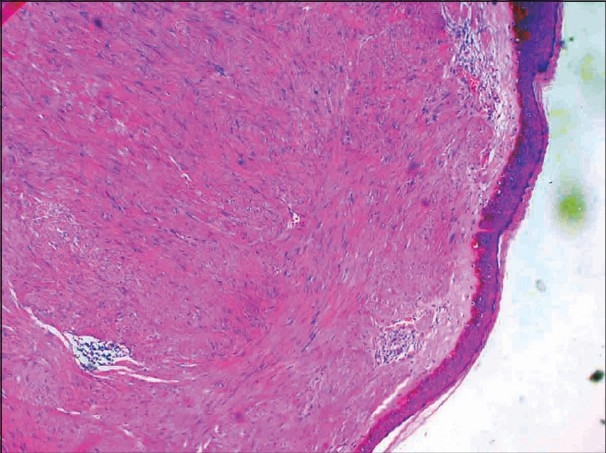
Histopathology: 4× view showing interlacing muscle bundles

**Figure 4:**
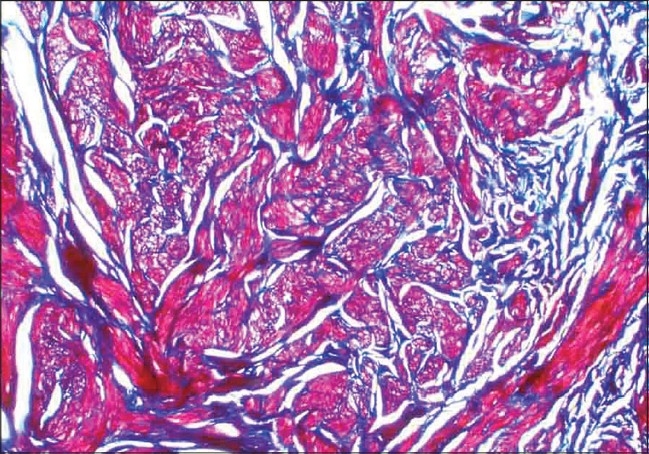
Histopathology: 40× view showing special stain (Masson's trichrome)

On examination, grouped, soft to firm, skin colored papules and nodules over the left side of the forehead, including the temple region [Figure 1], and extensive lesions over the back [Figure 2] were found. Lesions were not seen over any other area. On cold stimulation using ice, the patient reported severe pain over the lesions, especially over the forehead lesions. Systemic examination showed parameters that were within normal limits. A clinical diagnosis of multiple cutaneous leiomyoma was made, which was confirmed by a skin biopsy [Figure 3, Figure 4]. Based on the presence of segmental lesions on the forehead and the extensive lesions over the back, the case was labeled as a type 2 segmental manifestation of cutaneous leiomyoma. The patient was evaluated for other systemic involvement, and all investigations including blood and imaging studies were within normal limits.

The large number of lesions made surgical removal or CO_2_ lasers unfeasible. The basic aim was to do something for relieving the patient's pain.

The patient was initially started on Nifedipine 10mg three times a day, along with Hyoscine butyl bromide tablets 10mg twice a day. After two weeks, the patient reported significant reduction of pain over the lesions. Nifedipine was stopped and the patient was advised to continue with hyoscine butyl bromide 10mg twice a day on alternate days. After one more week, he was advised a single 10mg tablet of hysocine butyl bromide, twice a week. The patient had been under follow up for two months since and has been totally pain free during the period, even when exposed to cold. The patient had no problems in tolerating the pulsed dose of the drug. There was no history of any dry mouth, blurring of visions, constipation, or urinary difficulty.

## Discussion

Cutaneous leiomyomas are rare, benign tumors of the skin, which are present in multiple disseminated, segmental or solitary forms. The pathogenesis of segmental cutaneous leiomyomatosis is not yet fully known.

Two types of segmental manifestation of the autosomal dominantly inherited disease are postulated. Type 1 reflects heterozygosity for the underlying mutation, with a clinical picture similar to that in a non-mosaic phenotype. In type 2, loss of heterozygosity leads to homo- or hemizygosity, with a pronounced segmental manifestation of lesions in the affected segment.[1,2] Though the exact molecular etiopathogenesis of multiple cutaneous leiomyomas is not known, recent studies have demonstrated the involvement of a classical tumor suppressor gene encoding fumarate hydratase,[3,4] in the pathogenesis of multiple leiomyomas.

The pathogenesis of pain associated with these lesions is not clear. Various theories have been put forward, including pressure on nerve fibers and abnormal muscle contraction. It is also known that the excitation of the arrector pili muscle occurs via the sympathetic nervous system. Norepinephrine, secreted by postganglionic nerve fibers, activates the alpha-receptors of the muscle. Muscle contraction ensues; this is triggered by the influx of ions, most specifically calcium. Understanding this basic physiologic process may be relevant to the medical treatment of symptomatic leiomyomas.[5]

Surgical excision is often advocated as a treatment option when dealing with either solitary leiomyomas, or when the total number of lesions is only a few. Other modalities include - CO_2_ lasers and Cryosurgery.[6] Various pharmacological options have been tried for alleviating the pain associated with cutaneous leiomyomas. These include calcium channel blockers like nifedipine,[7] phenoxybenzamine,[8] doxazocine,[9] gabapentin,[10] and topical 9% hyoscine hydrobromide.[8]

In the case in study too, a trial with systemic Hyoscine, under the assumption that an agent like hyoscine may reduce the pain associated with the lesions by inducing muscle relaxation, was tried. Hyoscine is a relatively safe medication, though higher doses may produce uncomfortable anti-cholinergic effects like drying of mouth and blurring of vision. Pharmacological studies have revealed that hyoscine butylbromide is an anticholinergic drug, with a high affinity for muscarinic receptors located on the smooth-muscle cells of the gastrointestinal tract. Its anticholinergic action exerts a smooth-muscle relaxing/spasmolytic effect. Blockade of the muscarinic receptors in the GI tract is the basis for its use in the treatment of abdominal pain, secondary to cramping. Hyoscine butylbromide also binds to nicotinic receptors, which induces a ganglion-blocking effect.[11,12]

Several pharmacokinetic studies in humans have consistently demonstrated the low systemic availability of hyoscine butylbromide after oral administration, with plasma concentrations of the drug generally being below the limit of quantitation. The bioavailability of hyoscine butylbromide, estimated from renal excretion, was generally <1%. However, because of its high tissue affinity for muscarinic receptors, hyoscine butylbromide remains available at the site of action in the intestine and exerts a local spasmolytic effect.[11,12]

Several studies have evaluated the efficacy and safety of oral or rectal hyoscine butylbromide. Hyoscine butylbromide was considered beneficial in all of these trials, which supports its use in the treatment of abdominal pain caused by cramping. Hyoscine butylbromide is barely absorbed and detectable in the blood and does not penetrate the blood-brain barrier, and is, therefore, generally well tolerated. Few adverse events have been reported; in particular, no significant increases in the incidence of anticholinergic-related adverse effects have been observed.[11,12]

In our patient, effective control of pain was obtained by intermittent, pulsed administration of hyoscine, which also ensured that the drug did not produce any significant side-effect. There are no definite reports suggesting the use/dosage of oral hyoscine in dermatological conditions like cutaneous leiomyoma. Longer and larger trials are probably warranted to assess whether pulsed administration of muscle relaxants can be effective in combating the pain associated with cutaneous leiomyomas.
